# Epilepsy-related functional brain network alterations are already present at an early age in the GAERS rat model of genetic absence epilepsy

**DOI:** 10.3389/fneur.2024.1355862

**Published:** 2024-03-11

**Authors:** Lydia Wachsmuth, Leo Hebbelmann, Jutta Prade, Laura C. Kohnert, Henriette Lambers, Annika Lüttjohann, Thomas Budde, Andreas Hess, Cornelius Faber

**Affiliations:** ^1^Clinic of Radiology, University of Münster, Münster, Germany; ^2^Department of Experimental and Clinical Pharmacology and Toxicology, Friedrich-Alexander-University Erlangen-Nürnberg, Erlangen, Germany; ^3^Institute of Physiology I, University of Münster, Münster, Germany; ^4^Department of Neuroradiology, University Hospital Erlangen, Friedrich-Alexander-University Erlangen-Nürnberg, Erlangen, Germany; ^5^FAU NeW – Research Center for New Bioactive Compounds, Friedrich-Alexander-University Erlangen-Nürnberg, Erlangen, Germany

**Keywords:** GAERS, absence epilepsy, graph theory, functional connectivity, rs-fMRI, MEMRI, deformation-based morphometry, spike–wave-discharges

## Abstract

**Introduction:**

Genetic Absence Epilepsy Rats from Strasbourg (GAERS) represent a model of genetic generalized epilepsy. The present longitudinal study in GAERS and age-matched non-epileptic controls (NEC) aimed to characterize the epileptic brain network using two functional measures, resting state-functional magnetic resonance imaging (rs-fMRI) and manganese-enhanced MRI (MEMRI) combined with morphometry, and to investigate potential brain network alterations, following long-term seizure activity.

**Methods:**

Repeated rs-fMRI measurements at 9.4 T between 3 and 8 months of age were combined with MEMRI at the final time point of the study. We used graph theory analysis to infer community structure and global and local network parameters from rs-fMRI data and compared them to brain region-wise manganese accumulation patterns and deformation-based morphometry (DBM).

**Results:**

Functional connectivity (FC) was generally higher in GAERS when compared to NEC. Global network parameters and community structure were similar in NEC and GAERS, suggesting efficiently functioning networks in both strains. No progressive FC changes were observed in epileptic animals. Network-based statistics (NBS) revealed stronger FC within the cortical community, including regions of association and sensorimotor cortex, and with basal ganglia and limbic regions in GAERS, irrespective of age. Higher manganese accumulation in GAERS than in NEC was observed at 8 months of age, consistent with higher overall rs-FC, particularly in sensorimotor cortex and association cortex regions. Functional measures showed less similarity in subcortical regions. Whole brain volumes of 8 months-old GAERS were higher when compared to age-matched NEC, and DBM revealed increased volumes of several association and sensorimotor cortex regions and of the thalamus.

**Discussion:**

rs-fMRI, MEMRI, and volumetric data collectively suggest the significance of cortical networks in GAERS, which correlates with an increased fronto-central connectivity in childhood absence epilepsy (CAE). Our findings also verify involvement of basal ganglia and limbic regions. Epilepsy-related network alterations are already present in juvenile animals. Consequently, this early condition seems to play a greater role in dynamic brain function than chronic absence seizures.

## Introduction

Epilepsies have long been classified as neurological disorders that can affect the entire brain network (1). Noninvasive techniques, including electroencephalography (EEG), magnetoencephalography (MEG) and resting state-functional magnetic resonance imaging (rs-fMRI) are well-established diagnostic tools for individual assessment and systematic clinical research. Graph theory is an optional analysis strategy that is increasingly utilized for exploring brain network characteristics (2, 3).

The majority of clinical studies focus on temporal lobe epilepsy (TLE), which is the most common type of epilepsy (3, 4). TLE is characterized by convulsive seizures that arise from brain lesions caused by stroke, trauma, or tumor growth. Seizure activity, in particular prolonged seizures (status epilepticus) contribute to further neuronal damage (5). Absence epilepsy, on the other hand, is characterized by transient impairment of consciousness, brief interruption of ongoing activity, and momentary unresponsiveness to the environment. Absence seizures are typically not linked to local macroscopic lesions in the brain, leading absence epilepsies to be regarded as a benign syndrome (6). However, idiopathic generalized epilepsies (IGE) have been associated with abnormal behavior, cognitive impairment, including attention and memory deficits, language problems, and mood disorders (7). Because absence epilepsy and its comorbid symptoms mainly affect children and are less severe than convulsive seizures, ethical considerations make clinical studies, especially longitudinal assessments in humans [e.g., (8)], scarce. Furthermore, only few clinical reports relate observed network characteristics to duration of disease.

Standardized study designs can be utilized when working with animal models. Present knowledge is obtained from a wide spectrum of experimental techniques. Two-photon microscopy or local field potential (LFP) recordings can be used to assess neuronal activity, and can be combined with modulation of specific network components, for example using optogenetics or chemogenetics, to identify causal relationship between brain regions (9). Additionally, electrical recordings in awake animals are well-established. Permanent headmounts enable extended recordings and repeated recording sessions. While challenging, awake MRI in rodents is also possible (10). However, stress levels during the relatively long and noisy MR examinations may pose a serious obstacle since absence seizures typically occur in a relaxed behavioral state rather than during arousal. Pharmacological restraint, using a low dose of isoflurane, either alone or in combination with medetomidine, has been established for rs-fMRI studies in naïve rodents (11, 12). However, isoflurane suppresses seizures and only allows for investigating functional connectivity (FC) of epileptic animals in a seizure-free state. Investigating brain networks during seizures has been successfully performed under Neurolept sedation which preserves seizure occurrence [e.g., (13, 14)].

In two widely accepted, spontaneous rat models of absence epilepsy: the Genetic Absence Epilepsy Rats from Strasbourg (GAERS) and the Wistar Albino Glaxo/from Rijswijk (WAG/Rij) (10, 15–19) preclinical simultaneous EEG-fMRI studies have identified an epilepsy-specific, bilateral core brain network. In adult animals, this core network comprises somatosensory cortex and related thalamic regions during seizure initiation, generalizing across motor cortex and association cortex during seizures. Basal ganglia regions serve as remote seizure control. The contribution of limbic structures has been noted. These observations are consistent with analysis of rodent electrophysiologic data (20, 21).

The present longitudinal study used GAERS and age-matched non-epileptic controls (NEC) and aimed to further characterize the epileptic brain network using two functional measures, rs-fMRI and manganese-enhanced MRI (MEMRI). We started with 3 months-old rats when 100% seizure prevalence in GAERS was expected and performed monthly rs-fMRI examinations up to 8 months of age under low-dose Isoflurane anesthesia, i.e., in a seizure-free state. GAERS experience frequent non-convulsive seizures during 10–15% of their time in quiet wakefulness. We were particularly interested whether long-term aberrant brain activity or comorbidities associated with such a pathology would affect brain networks. After the final time point of the longitudinal rs-fMRI investigation at 8 months of age, we performed MEMRI as an alternative approach of evaluating FC. Finally, volumetric differences were assessed through deformation-based morphometry (DBM) on T1-weighted (T1w) 3D data between these 8 months-old GAERS and NEC rats. This approach provided readout of the effects of brain activity over different periods. Temporal synchronicity of regional brain activity (as probed by rs-fMRI) and volume changes (as probed by DBM) have been influenced over the previous life span. MEMRI, on the other hand, only represents differences in regional neuronal activity accumulated during the preceding phase of manganese application (7 days) and is not influenced by anesthesia, which is only necessary during the MRI acquisition.

## Material and methods

### Animals

**Figure 1:**
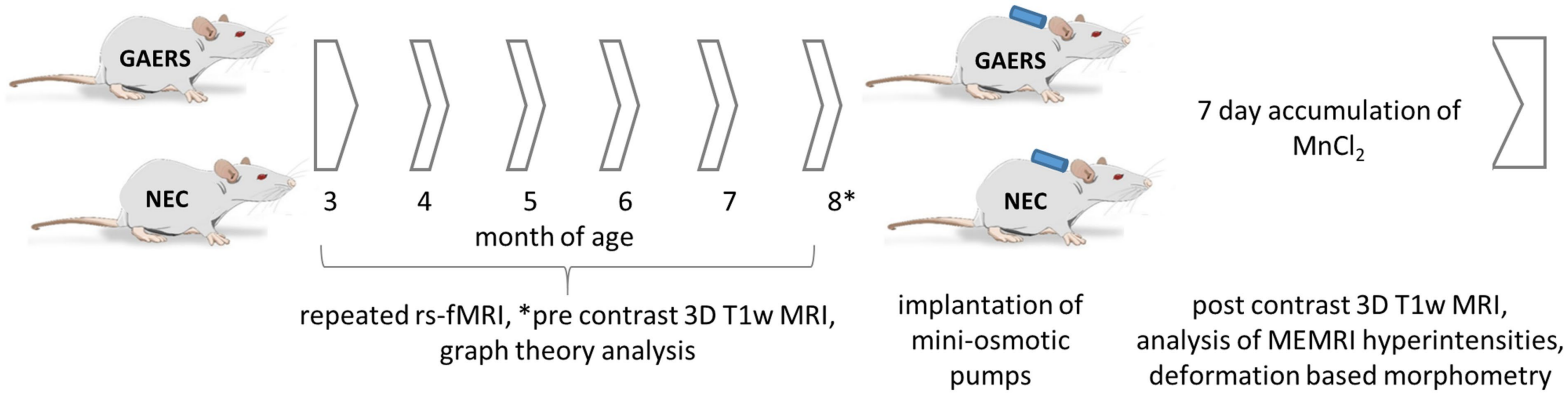
Study design. 12 GAERS and NEC, each, were subjected to repeated rs-fMRI examinations at 3, 4, 5, 6, 7, and 8 months of age under low dose isoflurane anesthesia. Graph theory analysis was applied to analyze longitudinal rs-fMRI data. At 8 months of age mini-osmotic pumps prefilled with MnCl_2_x4H_2_O were implanted. Before and after 7 days of continuous manganese application, high-resolution 3D T1w-MRI were acquired. Manganese accumulation was assessed by brain region-wise analysis of signal intensities in T1-weighted images. Regional volume differences between GAERS and NEC were retrieved from data registration.

**Figure 2:**
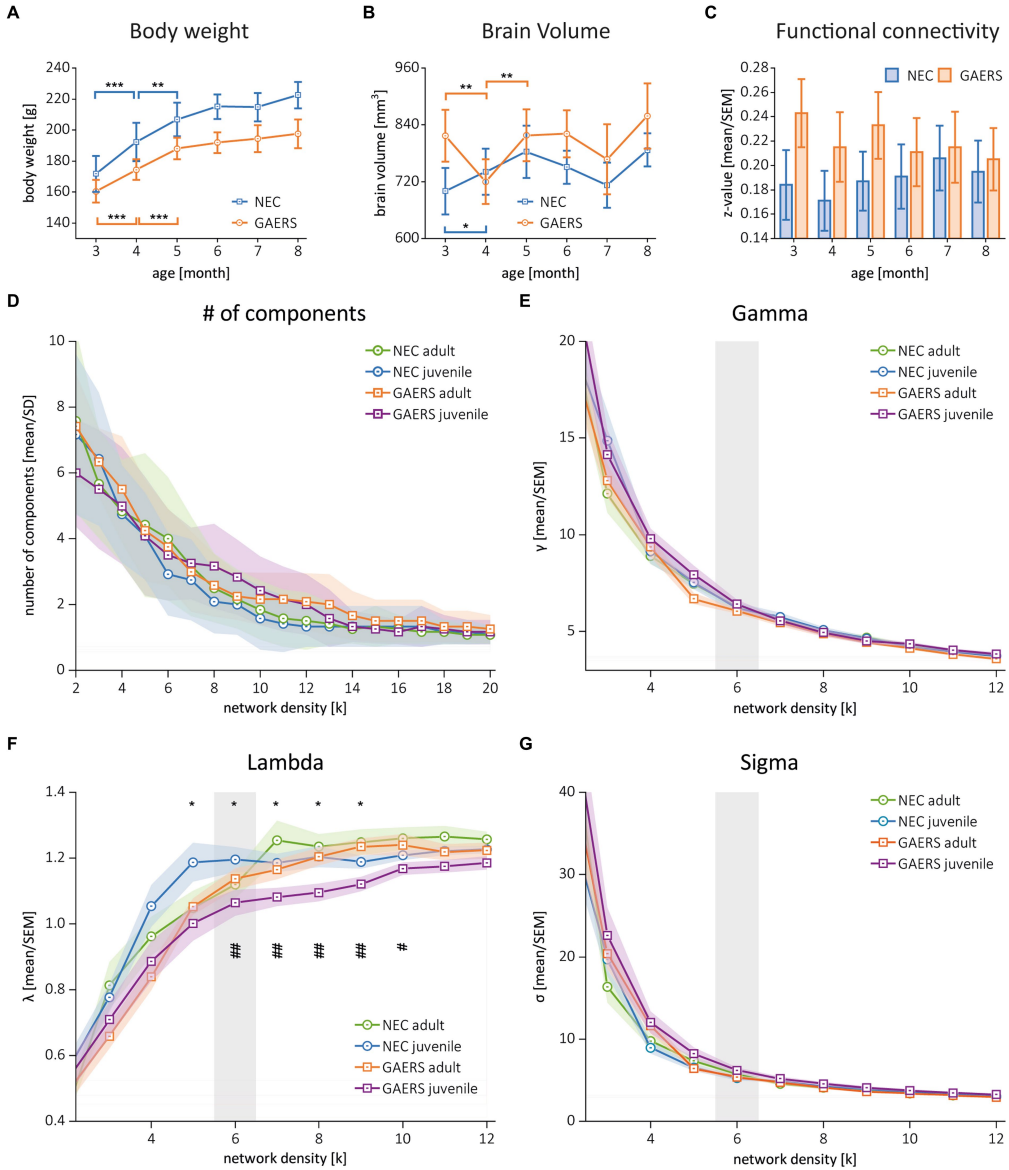
Global parameters. **(A)** Body weights of GAERS (blue) and NEC (orange) significantly increased between 3 and 5 months of age, followed by a plateau from 6 months and onwards. **(B)** Label mask volumes from rs-fMRI analysis with MagnAn were taken as rough estimate for brain volumes. Asterisks indicate significant differences between time points (rm ANOVA followed by posthoc Student’s *t*-test, *p < 0.05, **p < 0.01. and ***p < 0.001). **(C)** Mean correlation coefficients (*z*-transformed) of GAERS and NEC over time. Variance analysis with rm ANOVA revealed significant higher FC in GAERS when compared to NEC, but no effect of age (Table 1). **(D)** Number of components and **(E–G)** the global parameters clustering coefficient (gamma), shortest path length (lambda), and small world index (sigma) of juvenile and adult GAERS and NEC, respectively, plotted vs. network density *k*. Please note that *x*-axis in **E–G** only show network density up to *k* = 12. Asterisks **(F)** indicate significant differences of global parameter lambda between juvenile strains (*p < 0.05, unpaired Student’s *t*-test). Hash keys indicate significant differences between juvenile and adult GAERS (#*p* < 0.05 and ##*p* < 0.01, paired Student’s *t*-test). Grey bars highlight density threshold of *k* = 6, which was used for subsequent analyses. All graphs show mean and standard error of the mean (SEM).

Experiments were performed in line with ARRIVE guide lines with female GAERS (*n* = 12) and NEC (*n* = 12) from the breeding colony of the Institute of Physiology I, University of Münster (animal use protocol 84-02.04.2015.A427, Landesamt für Natur, Umwelt und Verbraucherschutz Nordrhein-Westfalen). Both strains have the same genetic background: they were outbred from Wistar rats, one exhibiting the EEG and behavioural pattern of absence epilepsy (GAERS) and the other being seizure-free (NEC) (22). In the GAERS, the penetrance and frequency of spike–wave discharges (SWD) does not differ as a function of sex (23). Rats were housed in groups of two to four animals at 21°C, controlled relative humidity of 45–65%, a 12/12 h light/dark cycle with unlimited access to food and water. In order to enrich animal’s environment, we used 13 inches high cages with a second level and offered red-transparent plexiglas houses and aspen gnawing sticks. Monthly longitudinal MRI examinations were performed between ages of 3 to 8 months (6 instances). Study design is outlined in Figure 1. Prior to each examination, animal body weights were measured (Figure 2A).

### Resting state-fMRI

#### Animal preparation

Animals were anesthetized with 5% Isoflurane (Baxter vet., Unterschleißheim, Germany) and positioned in the animal cradle with head fixation via ear plugs and bite bar. During MRI examinations a gas mixture of 75% air and 25% oxygen with 1.1–1.2% Isoflurane was supplied via a nose cone. Respiration rate and rectal body temperature were continuously monitored and strictly kept in the physiological range (36.5–37.5°C, 60–90 breaths per minute) by adjusting cradle temperature and Isoflurane dose. We paid particular attention to keep the time between initiation of anesthesia and functional scans as short as possible (20–30 min).

#### Acquisition

rs-fMRI data were acquired at 9.4 T (Bruker Biospin MRI GmbH, Ettlingen, Germany) using Paravision 5.1 with a 3-element rat brain surface coil (Rapid Biomedical, Rimpar, Germany) applying a gradient echo-echo planar imaging sequence (GE-EPI) with repetition time TR 1 s, echo time TE 18 ms, resolution 0.33 × 0.35 mm^2^, slice thickness 1.2 mm, 12 contiguous axial slices and 30 min scan time per data set. Slice 5 from caudal was placed across the anterior commissure. Saturation slices were placed around the brain to suppress signal from outside of the brain. Local shimming was accomplished using Mapshim in a volume of 12 × 14 × 15 mm^3^ (anterior–posterior × left–right × rostro–caudal).

#### Preprocessing

Raw data were realigned and resliced using SPM12 (http://www.fil.ion.ucl.ac.uk/spm, based on MATLAB 2018b, Mathworks, Natick, MA, United States). The first 15 min of the 30 min raw data were used. In case any shift in rat head position was detected, continuous 15 min epochs with stable translation (<0.2 mm) and rotation (<0.3°) parameters were selected from 30 min data sets. For data quality assurance, a bilateral cortical ROI in slice 9 was used to estimate temporal Signal-to-Noise-Ratio (tSNR) (GAERS 57 ± 10, NEC 56 ± 11). Subsequent processing was done using MagnAn (Biocom, Uttenreuth, Germany), a MRI image analysis software based on IDL (^©^2020 Harris Geospatial Solutions, Inc.). 2D Gaussian smoothing with a kernel size of 3 × 3 pixel, full width half max 0.6 mm was applied. Time series were low pass filtered using a Fourier-filter of 0.1 Hz. Brains were manually masked and the global signal mean was regressed out. A template of 80 brain regions (24) (Supplementary Table S1, referred to as MagnAn atlas) based on the Paxinos and Watson rat brain atlas (25) was semi-automatically registered to each individual dataset (with the Regibox tool provided in MagnAn followed by manual correction). The label mask volumes were taken as a rough estimate for brain volumes (Figure 2B). 8 months rs-fMRI data was additionally analyzed using a template with 114 brain regions based on the RatSigma atlas (26) in order to allow for region-wise comparison with high-resolution MEMRI data acquired at the final time point of the study (see below). The RatSigma atlas subdivides cortex and hippocampus into many subregions but provides only very limited subcategories for the subcortical brain (Supplementary Table S1).

#### Network construction

Network analysis was performed using MagnAn with a multi seed regions approach (MSRA) (24). In brief, seed regions were automatically defined in the center of mass of each brain region. The average time course of each seed region was correlated with every voxel time course within the brain and the resulting correlation maps were thresholded using the Benjamini–Yekutieli false discovery rate (FDR), implemented in MagnAn (*q* = 0.05, *n* = 6 time points). The average Pearson’s correlation coefficient *r* of all target voxels per brain region was used to define the connectivity strength to the respective seed region. This procedure was repeated for every brain region resulting in an asymmetric correlation matrix 80 × 80 (MagnAn atlas) and 114 × 114 (RatSigma atlas) per resting-state scan. Pearson’s *r*-values were transformed to Fisher’s *z*-values to provide normal distribution and only positive correlations were considered further. *Z*-values of correlation coefficients were initially compared for each time point (Figure 2C and Table 1).

**Table 1:** Group comparisons.

	Factor	df	*F*	*p*
**(A)** FC (*z*-values)				
GAERS vs. NEC	Strain	1	13.910	0.001**
6 time points	Time point	5	2.30	0.085
Interaction	Strain * time point	5	1.767	0.129
**(B)** Body weight				
GAERS vs. NEC	Strain	1	22.589	<0.001***
6 time points	Time point	5	182.477	<0.001***
Interaction	Strain * time point	5	3.545	<0.01**
**(C)** Brain volume				
GAERS vs. NEC	Strain	1	64.722	<0.001***
6 time points	Time point	5	19.921	<0.001***
Interaction	Strain * time point	5	4.662	<0.01**
**(D)** FC (*z*-values)				
GAERS vs. NEC	Strain	1	11.468	0.003**
Juvenile vs. adult	Age group	1	0.271	0.608
Interaction	Strain * age group	1	1.225	0.275

Summary of rm ANOVA comparing **(A)** FC (*z*-values), **(B)** body weights, and **(C)** brain volumes per strain and time point. **(D)** FC after combining 3, 4, and 5 months data in a juvenile group and 6, 7, and 8 months data in an adult group.

For subsequent analyses, we combined data from 3–5 months (juvenile) and 6–8 months (adult) based on animal body weights. Averaged group correlation matrices of juvenile and adult GAERS and NEC are displayed in Supplementary Figure S1. The clearly visible diagonals, representing the interhemispheric connections between regions, indicate that the analysis established meaningful functional connections, despite anatomical distance. Robust interhemispheric connectivity was observed at a significance level of *p* < 0.05, FDR corrected, between primary sensory hindlimb cortices for both NEC (*r* = 0.389 ± 0.138) and GAERS (*r* = 0.463 ± 0.173). In contrast, there was low intrahemispheric connectivity strength between hindlimb cortex and cingulate cortex (NEC 0.291 ± 0.111, GAERS 0.332 ± 0.124). These findings confirm specificity of our data to known functional connectivities (27).

#### Global network parameters

The global network parameters clustering coefficient, shortest path length, and small world index (28, 29) were calculated using MagnAn for a range of network densities (*k* = 2–20). Metrics were normalized by comparison to 1,000 random networks with equivalent nodes and edges count. Network parameters are dependent on the network density *k*, which is the ratio of the actual number of edges to the total number of potential edges between all nodes in the network. The clustering coefficient measures the fraction of neighbors of a node that are neighbors of each other. The global clustering coefficient *γ* is the average of all local clustering coefficients. The global shortest path length *λ* indicates the efficiency of the overall information flux within the network and is defined by the average number of edges needed to travel from one node to another. The small world index *σ* is the ratio of the latter two metrics, *γ*/*λ*. Small worldness is greater than 1 for networks with tightly interconnected clusters of nodes, as seen in regular networks, but also for short path lengths between elements, as in random networks (29). As the network density increases, global network parameters become more similar. Global parameters were compared on group level with unpaired (GAERS vs. NEC) or paired (juvenile vs. adult) Student’s *t*-test. The network density of *k* = 6, representing the strongest 240 connections between brain regions (i.e. approximately 8% of all connections), was selected as the threshold for all further analyses, as it approximately corresponds to the inflection point in global parameter plots.

#### Local network parameters

Local network parameters were calculated using MagnAn. Degree, strength, clustering coefficient, shortest path length, betweenness and hub score were determined for each node and data set. The degree represents the number of connections associated with a single node. The strength is calculated as the sum of link weights of a node. A high hub score indicates importance of brain regions with above-average high number of connections (degree), a high level of betweenness centrality and short average path length (30). Local node parameter data did not pass Shapiro Wilk-test (*p* > 0.05) for normal distribution. Group comparisons were performed with the Kruskal–Wallis-test (IBM SPSS statistics 25, Table 2). Obvious group differences in local node parameters were then tested for statistical significance by applying posthoc Mann Whitney U-test with correction for multiple comparisons.

**Table 2:** Group comparisons.

(A)		Strength		Degree		CC		PL		BW		HS	
Factor	df	H	*p*	H	*p*	H	*p*	H	*p*	H	*p*	H	*p*
Hemisphere	1	2.503	0.114	2.804	0.094	1.231	0.267	1.161	0.281	3.122	0.077	3.223	0.073
Strain	1	9.967	0.002**	0.017	0.897	2.888	0.089	42.434	<0.001***	0.76	0.383	0.938	0.333
Age	1	1.805	0.179	2.357	0.125	2.856	0.091	0.143	0.705	0.487	0.485	1.517	0.218
Group	3	14.605	0.002**	2.713	0.438	7.171	0.067	58.289	<0.001***	2.926	0.403	2.775	0.428

(B)	Strength			Path length		
Factor	df	H	*p*	df	H	*p*
Juvenile, GAERS vs. NEC	1	7.020	0.008**	1	24.865	<0.0001***
Adult, GAERS vs. NEC	1	0.800	0.371	1	3.461	0.063

Summary of non-parametric testing (Kruskal–Wallis-test) of local node parameters at *k* = 6. **(A)** First 38 bilateral brain regions were tested for interhemispheric differences. Then variance analysis of effects of strain (GAERS and NEC), age (juvenile and adult) and group (juvenile GAERS and NEC and adult GAERS and NEC, respectively) was applied to 42 brain regions. **(B)** Interaction between strain and age was significant for the local node parameters strength and path length in the juvenile groups. CC, correlation coefficient; PL, shortest path length; BW, betweenness; HS, hub score.

#### Network topology

**Figure 3:**
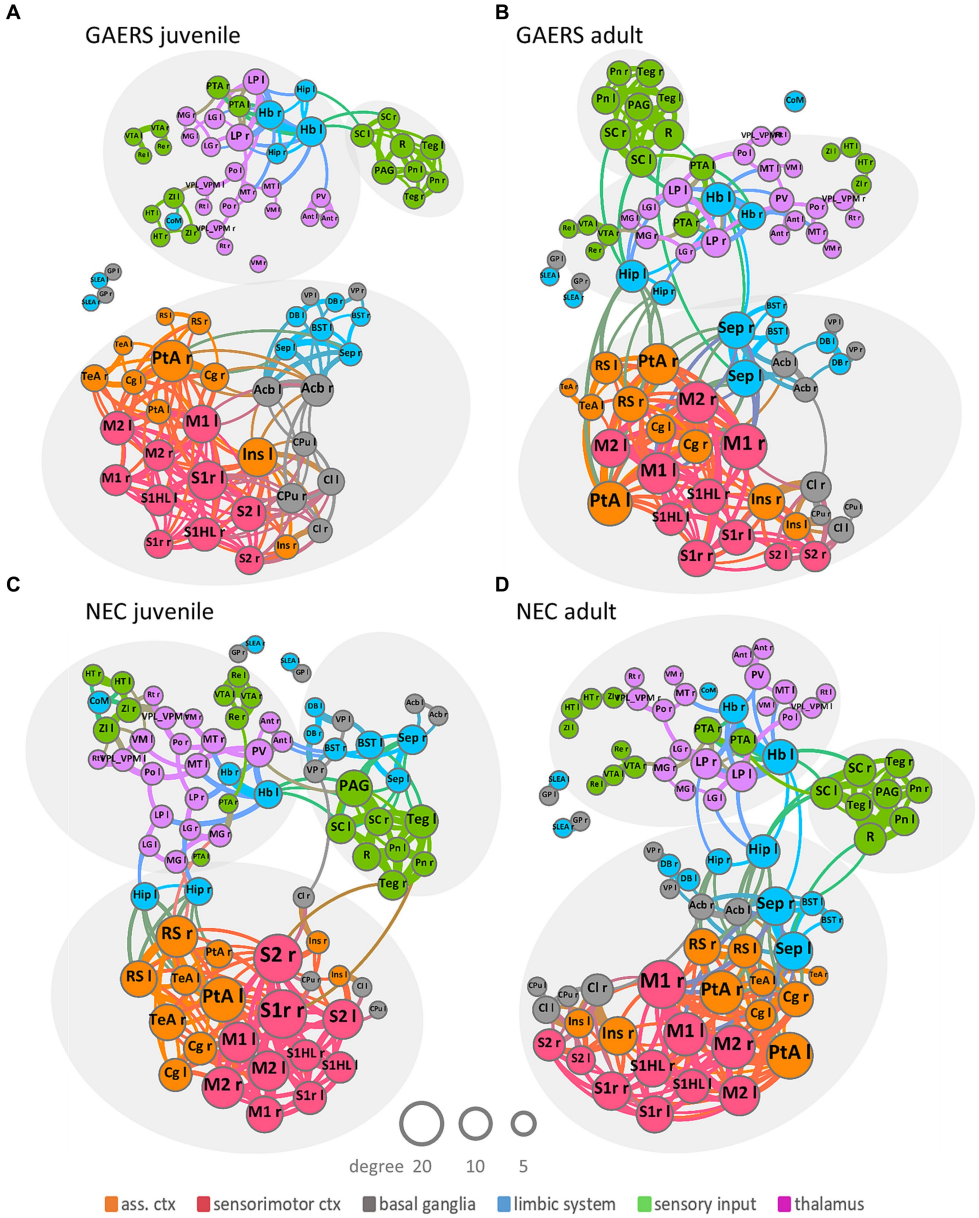
Network topology. Group-averaged force-based network plots of juvenile and adult GAERS **(A,B)** and NEC **(C,D)**, respectively, at a network density of *k* = 6. Nodes represent brain regions and edges connections between them. Arrangement and closeness of nodes takes correlation strength between brain regions into account. Brain networks subdivided into three major communities, indicated by light grey ellipses, irrespective of strain and age: one cortical community, comprising association cortex (orange) and sensorimotor cortex (red) mingling with basal ganglia (grey) and some limbic regions (blue); one sensory input community (green) colocalized with limbic region periaqueductal grey (blue) and one subcortical community comprising thalamic (pink), sensory input (green) and limbic regions (blue). Node and label size scale with degree and edge thickness represents the correlation strength between connected brain regions. Nodes are color-coded according to affiliation with functional group. Abbreviations of brain regions can be found in Supplementary Table S1.

**Figure 4:**
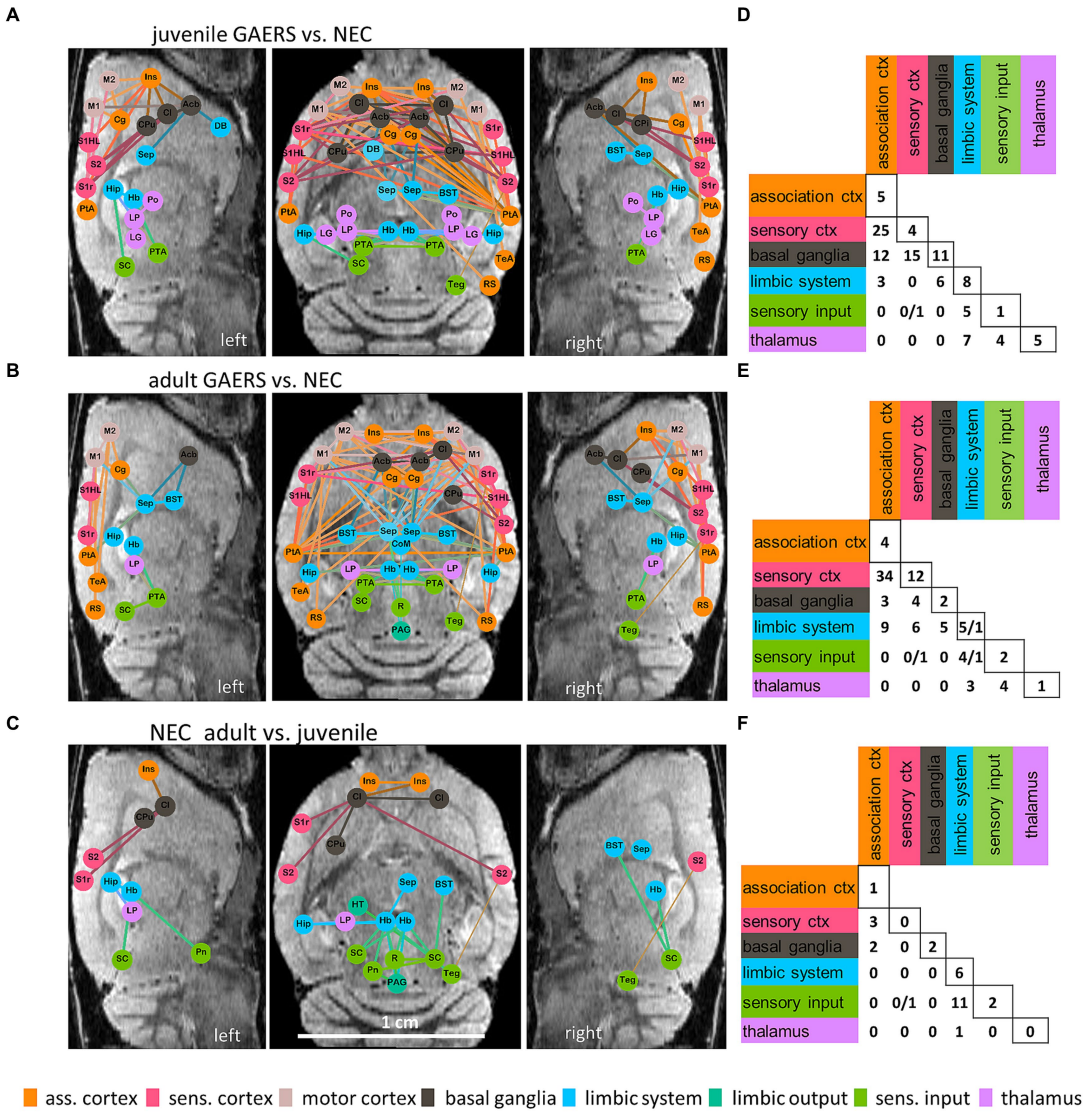
Network based statistics. NBS at *k* = 6 revealed significant differences between brain networks of GAERS and NEC in both, the juvenile **(A,D)** and the adult **(B,E)** groups, and between juvenile and adult NEC **(C,F)**. Graphical layouts **(A–C)** are overlaid, in approximate anatomical position, on one representative left and right sagittal and one axial MR image (scale bar in grey). Only nodes with significantly altered connections are shown. Colors encode affiliation with the functional group. The colors of the edges are a combination of the colors of the connected nodes. Statistically stronger connections in GAERS compared to NEC **(A,B)** and in adult compared to juvenile NEC **(C)** are represented by thick lines, thin lines represent statistically weaker connections for the respective comparison. Matrices **(D–F)** summarize the numbers of significantly different connections between brain regions per functional group (please note, sensory cortex and motor cortex as well as limbic system and limbic output were combined) when comparing GAERS with NEC **(D,E)** and adult with juvenile NEC **(F)**. If two numbers are shown in one cell, the first represents the number of stronger, the second the number of weaker connections for the respective comparison. Group permutations: 1,000, alpha: *p* < 0.05.

Community structure was evaluated using Gephi, version 0.9.7, an open-source software for graph theoretical analysis (31). The algorithm (32) partitioned the rs-fMRI network into sub-communities of densely connected nodes, with nodes from distinct communities exhibiting sparse connections. To display the network (Figure 3), we used the force-based algorithm implemented in Gephi (33). Node labels identify anatomical brain regions. Color of nodes indicates affiliation with functional group. Magnitudes of degree were utilized to code node and label size in network depictions. Edge thickness was calibrated to reflect strength of correlation coefficients. Group differences in network connectivity between strains and age groups were assessed using network-based statistics (NBS), implemented in MagnAn. A homoscedastic Student’s *t*-test between FDR-corrected correlation matrices at *k* = 6 was performed and corrected for multiple testing by 1,000 times permutation (34). The identified significant component of functional connections that differed between GAERS and NEC, as well as between juvenile and adult NEC, is displayed as overlay on a set of representative midline axial and lateral sagittal T1w images (Figure 4).

### Manganese-enhanced MRI

#### Preparation

Within a week after the last rs-fMRI examination, mini-osmotic pumps (Alzet, Model 2001) were subcutaneously implanted in the shoulder region. The pumps had been prefilled with 200 μL MnCl_2_ × 4H_2_O (Sigma) in 0.4 M bicine buffer (Sigma), pH 7.4, to continuously deliver a cumulative dose of 150 mg/kg MnCl_2_ × 4H_2_O within 7 days at a rate of 1 μL/h. For surgery, animals were anesthetized with Isoflurane (initiation 5%/continuous dose 2.5% in 100% oxygen) and received buprenorphine (bolus s.c. 0.05 mg/kg, Temgesic, Reckitt Benckiser Healthcare UK Ltd., Hull, United Kingdom) 30 min before surgery. Rats tolerated subcutaneous pump implantations and 7d continuous manganese applications well.

#### Acquisition

We acquired T1w 3D FLASH scans prior to and at day 8 after pump implantation. Imaging parameters were as follows: TR/TE: 21/7.6 ms, FA: 10°, 3 × 3 × 2 cm^3^ FOV, 320 × 320 × 80 matrix, 94 × 94 × 250 μm^3^. We applied fat suppression and respiration trigger. Signal enhancement in the pituary gland and dentate gyrus confirmed manganese delivery via osmotic pumps in all animals.

#### Analysis

Brains were manually segmented from 3D T1w FLASH scans and registered on the template of the RatSigma atlas with 114 brain regions via ANTS (Advanced Normalization Tools [47], two stage build-template approach). Next, median smoothing with a 7 × 7 × 7 kernel was performed and a homoscedastic Student’s *t*-test was calculated between registered images of GAERS vs. NEC animals (*n* = 12, each) to compare signal intensities. On the resulting *t*-values a threshold-free cluster enhancement (TFCE) was applied and finally a multiple comparison correction (100 repetitions with random group assignment) was performed. Voxels were thresholded at 99.9th percentile *t*-value (*t* = 4.02420, corresponding to *p* = 0.00057). The resulting color map shows areas with more than 500 voxels exhibiting significant signal enhancement in GAERS compared to NEC, overlaid on the mean difference image.

Registration of high-resolution 3D T1w datasets additionally provided morphometric data. For analysis, the RatSigma atlas was transformed into each individual subjects’ space using the inverse of the deformation field per subject obtained in the above registration step. The average volumes of each brain structure in all GAERS and NEC were analyzed using a Student’s *t*-test to identify brain structures significantly differing in volume. The lack of difference in whole brain volumes pre and post manganese application (for NEC 1759 ± 34 mm^3^ (pre), 1767 ± 37 mm^3^ (post), Student’s paired *t*-test 0.088; and for GAERS 1821 ± 40 mm^3^ (pre), 1828 ± 36 mm^3^ (post), Student’s paired *t*-test *p* = 0.403) suggests that signal enhancement due to manganese accumulation did not significantly impact brain volume determination in T1w 3D data sets.

## Results

The present longitudinal rs-fMRI study started with 3 months old rats, an age at which GAERS have a seizure prevalence of 100% (22). We found general differences between epileptic and non-epileptic brain networks. Other than expected, no brain network alterations/adaptations were detected in GAERS over the ensuing 5 months of life.

The initial analysis of *z*-transformed correlation coefficients averaged by strain and examination time point (Figure 2C) showed generally higher FC for GAERS than for NEC (repeated measure (rm) ANOVA, *p* < 0.01; Table 1A). However, no substantial differences were observed with increasing age of animals. It was assumed that animals had fully matured at 6 months of age, and therefore, correlation matrices were averaged across 3, 4, and 5 months data and classified as juvenile NEC/GAERS, and averaged across 6, 7, and 8 months data and classified as adult NEC/GAERS. This grouping of data was based on the body weight time profile, which demonstrated an increase from 3 to 5 months of age, followed by a plateau from 6 months and onwards (Figure 2A; rm ANOVA Table 1B). Brain volumes (determined through data registration), also only increased significantly between 3 to 5 months of age (Figure 2B; rm ANOVA Table 1C). A similar age categorization had been applied in a recent longitudinal study in Long Evans rats (which also exhibit absence seizures) by Kozac et al. (35). The subsequent analyses were performed on these grouped data.

### Global network topology

The global network parameters clustering coefficient, shortest pathlength, and small world index became similar with increasing network density between all groups (Figures 2E–G). The similarity in global parameters suggested that both GAERS and NEC have similarly efficient brain networks. To identify potential differences, we chose a *k* = 6 (inflection point in Figures 2E–G), considering the 240 (that is the 8%) strongest connections as threshold for the construction of networks, the NBS, and the calculation of local node parameters.

### Community structure

Force-based network plots (Figure 3) at *k* = 6 were used to display network topologies of juvenile and adult GAERS and NEC. This graphic illustrates the 80 brain regions as nodes with the 240 strongest connections represented as edges. Arrangement and proximity of nodes takes correlation strength between brain regions into account. Node and label sizes scale with degree, which is defined as the number of connections per brain region, while edge thickness indicates the correlation strength between connected brain regions. Overall community structure was comparable in both age groups in GAERS (Figures 3A,B) and NEC (Figures 3C,D). Across all groups, most connections were formed between cortical regions. Brain networks subdivided into three major communities, indicated by light grey ellipses: One community was primarily cortical, with regions of sensorimotor cortex (red) and association cortex (orange) associated with basal ganglia (grey) and some limbic regions (blue). The second community was composed of regions from sensory input (green) and the limbic region periaqueductal gray (PAG, blue) and some basal ganglia regions in juvenile NEC. The third community included thalamic (pink), limbic (blue), and sensory input regions (green). In juvenile GAERS (Figure 3C), the cortical community was entirely separate from the exclusively subcortical communities. Even the limbic and basal ganglia regions, associated closely with the cortical community in all other groups, tended to form a small subcommunity in juvenile GAERS (to a lesser extent also observed in adult GAERS). The consistent gathering of respective left and right bilateral brain regions within the same community confirmed strong, preserved functional connectivity between the hemispheres.

### NBS

NBS at *k* = 6 identified notable differences of brain networks between strains in both age groups (Figures 4A,B,D,E), but only NEC showed significant differences with maturation (Figures 4C,F). Brain networks of GAERS formed stronger connections (thick lines) than age-matched NEC, while only few connections were significantly weaker (thin lines) (Figures 4A,B). When comparing juvenile groups (Figures 4A,D), NBS revealed 42 nodes involved in network changes in the epileptic brain, with 111 stronger and 1 weaker connections (component probability *p* < 0.001). When comparing adult groups (Figures 4B,E), 39 nodes were involved in 99 stronger and 3 weaker connections (component probability: *p* = 0.002). Overlaying nodes with statistically different connection strength on anatomical sagittal and coronal MR images (Figures 4A–C), highlights the enhanced FC of brain regions within and between hemispheres in epileptic animals. Irrespective of age, GAERS exhibited stronger connections, particularly among brain regions of association cortex (orange), sensory cortex (red) and motor cortex (rose) and with basal ganglia (grey) and limbic regions (blue). Similarly, thalamic regions (pink) were more strongly interconnected in GAERS when compared to NEC. Far less statistically significant differences were identified between juvenile and adult brain networks of rats of the same strain. For NEC (Figures 4C,F), there were 21 nodes identified, with 29 stronger and 1 weaker connection (component probability: *p* = 0.028). Adult NEC formed stronger connections within and between limbic (blue) and sensory input regions (green), and between cortical (red and orange) and basal ganglia (grey) regions. However, in GAERS, NBS did not indicate significant age-related differences (component size: 14 nodes, 14 connections; component probability: *p* = 0.182).

### Local node parameters

**Figure 5:**
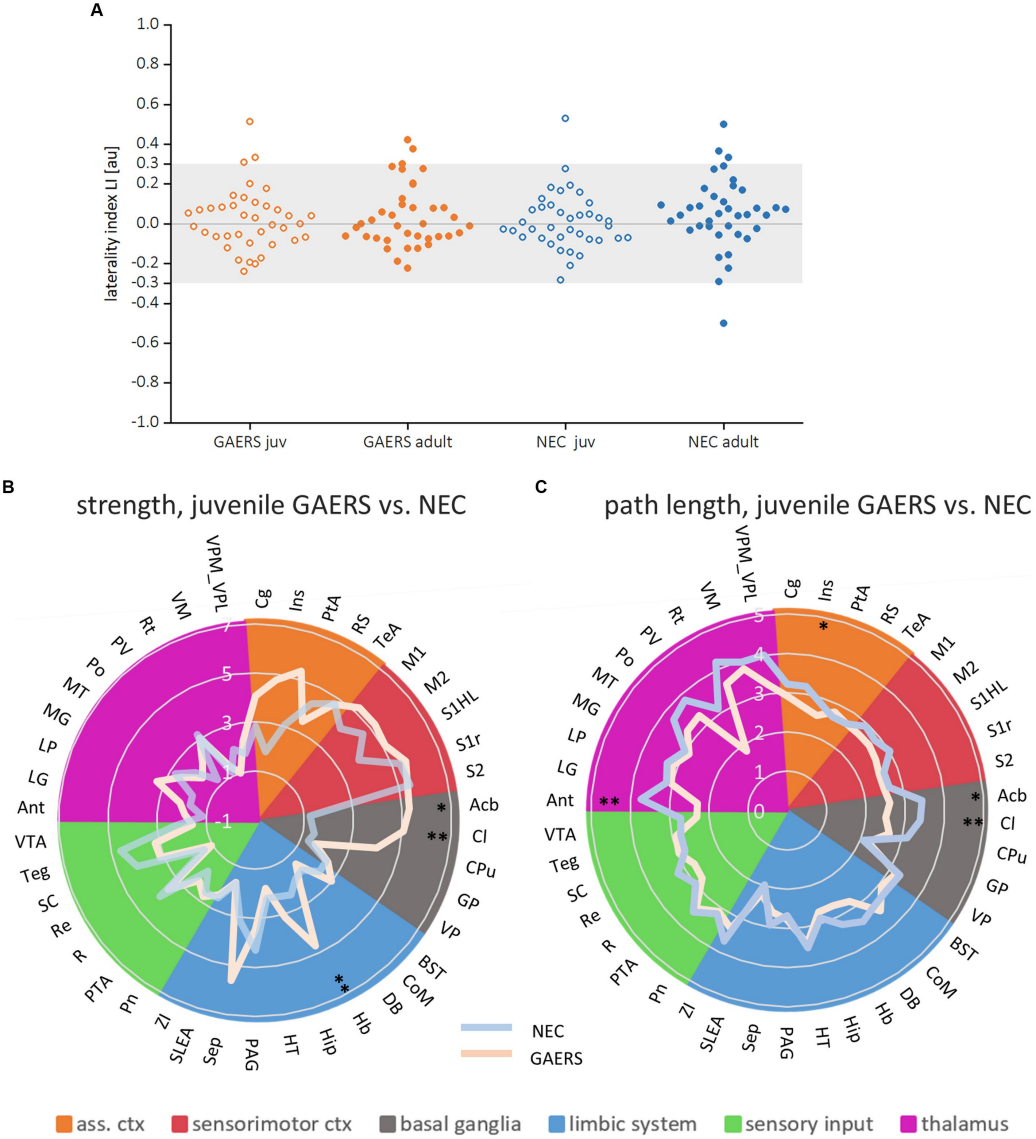
Local parameters. **(A)** Laterality indices of the local parameter degree for both juvenile and adult GAERS and NEC do not indicate hemispheric dominance. Dots represent brain regions, light grey areas indicate the laterality index (LI) threshold set at 0.3. Radar plots display strength **(B)** and path length **(C)** of juvenile GAERS (light blue) and NEC (light orange) averaged across hemispheres. Asterisks indicate significant differences between strains (Posthoc Mann Whitney U *p < 0.05 and **p < 0.01). List of abbreviations of brain regions can be found in Supplementary Table S1. Summary of results of non-parametric variance analysis are reported in Table 2.

Local node parameters were calculated in addition to the NBS to further pinpoint which brain regions were specifically engaged in the epileptic network. Across all groups, the top 20 regions in terms of degree rank were observed in both hemispheres in association cortex, sensorimotor cortex and limbic system (highlighted by bold colors in Supplementary Figures S2A,B). In all groups except juvenile GAERS, sensory input regions were also present in the top 20 regions of degree. Almost exclusively in GAERS, basal ganglia regions were among the top 20 in degree. The laterality index of brain region-wise degrees did not indicate hemispheric dominance (Figure 5A). Additionally, group comparisons did not reveal significant interhemispheric differences for any of the local node parameters between 38 bilateral brain regions (Table 2A). Kruskal–Wallis-Test revealed significant effects of strain (for strength and path length), but not of age. Significant interactions were found between groups for strength and path length, with a significant effect in juvenile groups (Table 2B). Radar plots illustrate the local node parameters strength (Figure 5B) and path length (Figure 5C) in juvenile rats, averaged by hemispheres. The posthoc analysis (Mann Whitney U-Test) of juvenile groups revealed significantly higher strengths of the basal ganglia regions nucleus accumbens (Acb) and claustrum (Cl), and the limbic region habenulum (Hb) in GAERS. Path length was significantly lower in the insular cortex (Ins) and in the basal ganglia regions Acb and Cl in juvenile GAERS. By closer examination of local parameters that were averaged over hemisphere and age (Supplementary Figure S3), posthoc testing revealed significantly higher strength (Supplementary Figure S3A) and degree (Supplementary Figure S3B) in GAERS in regions of the association and the sensorimotor cortex, cingulate cortex (Cg), parietal association cortex (PtA), Ins, and primary motor cortex (M1), as well as in the limbic region septum (Sep). In NEC, degree was higher in periaqueductal gray (PAG) and in the sensory input regions superior colliculus (SC) and tegmentum (Teg). In NEC, significantly higher clustering coefficients were observed (Supplementary Figure S3C) in the sensory input regions pretectal area (PTA) and pontine nucleus (Pn), and in paraventricular thalamic nucleus (PV). The association cortex regions PtA and Ins, sensory cortex of the hindlimb (S1HL) and the basal ganglia region Cl exhibited significantly lower path length (Supplementary Figure S3D) in GAERS when compared to NEC. A statistically significant higher betweenness (Supplementary Figure S3E) was observed in Acb and caudate putamen (Cpu). Secondary sensory cortex (S2) betweenness was higher in NEC. PtA, Acb, and Cpu showed a significantly higher hub score (Supplementary Figure S2F) in GAERS. In NEC, the sensory input regions Teg, SC, Pn and raphe nucleus (R) were identified as network hubs.

Differences in local network parameters supported the picture revealed by NBS, highlighting the segregation of cortical regions, and the prominent role of basal ganglia and limbic nodes in the epileptic network.

#### MEMRI

**Figure 6:**
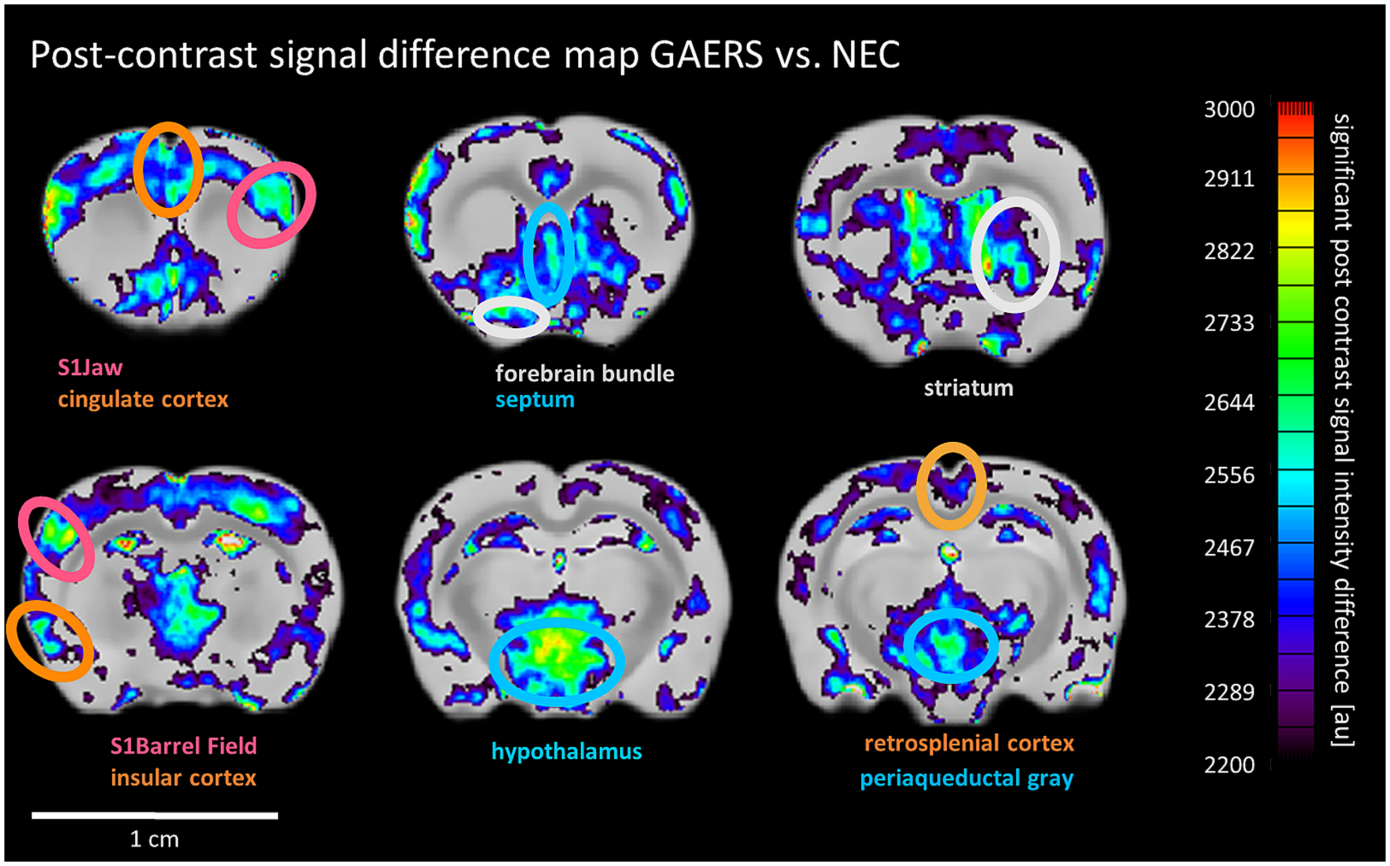
MEMRI post-contrast signal difference maps. Pixels with significantly higher post contrast signal intensity in GAERS than NEC are shown and overlaid onto the mean difference image. Ellipses highlight selected areas with significant vs. NEC’s signal enhancement. Color scale uses arbitrary units for signal intensity difference. Color of labels and ellipses indicate functional group affiliation.

Next, we investigated whether an alternative functional readout based on long-term accumulation of manganese over time in adult behaving animals after the rs-fMRI study’s last time point would yield results consistent with our rs-fMRI findings. In general, continuous manganese application over 7 days to 8 months-old rats of both strains resulted in non-uniform SI enhancement, particularly strong in the hippocampus, amygdala, and cortex (Supplementary Figure S4). Threshold-free cluster enhancement (TFCE) was applied to compare manganese accumulation pattern between strains. TFCE maps (Figure 6) show significantly higher signal intensity in GAERS in sensory cortex regions like barrel field and jaw. Additionally, significantly higher signal was evident in GAERS in the association cortex (retrosplenial, insular and cingulate cortex). Striatum and limbic regions, specifically the septum and hypothalamus also showed statistically significant signal increase.

**Figure 7:**
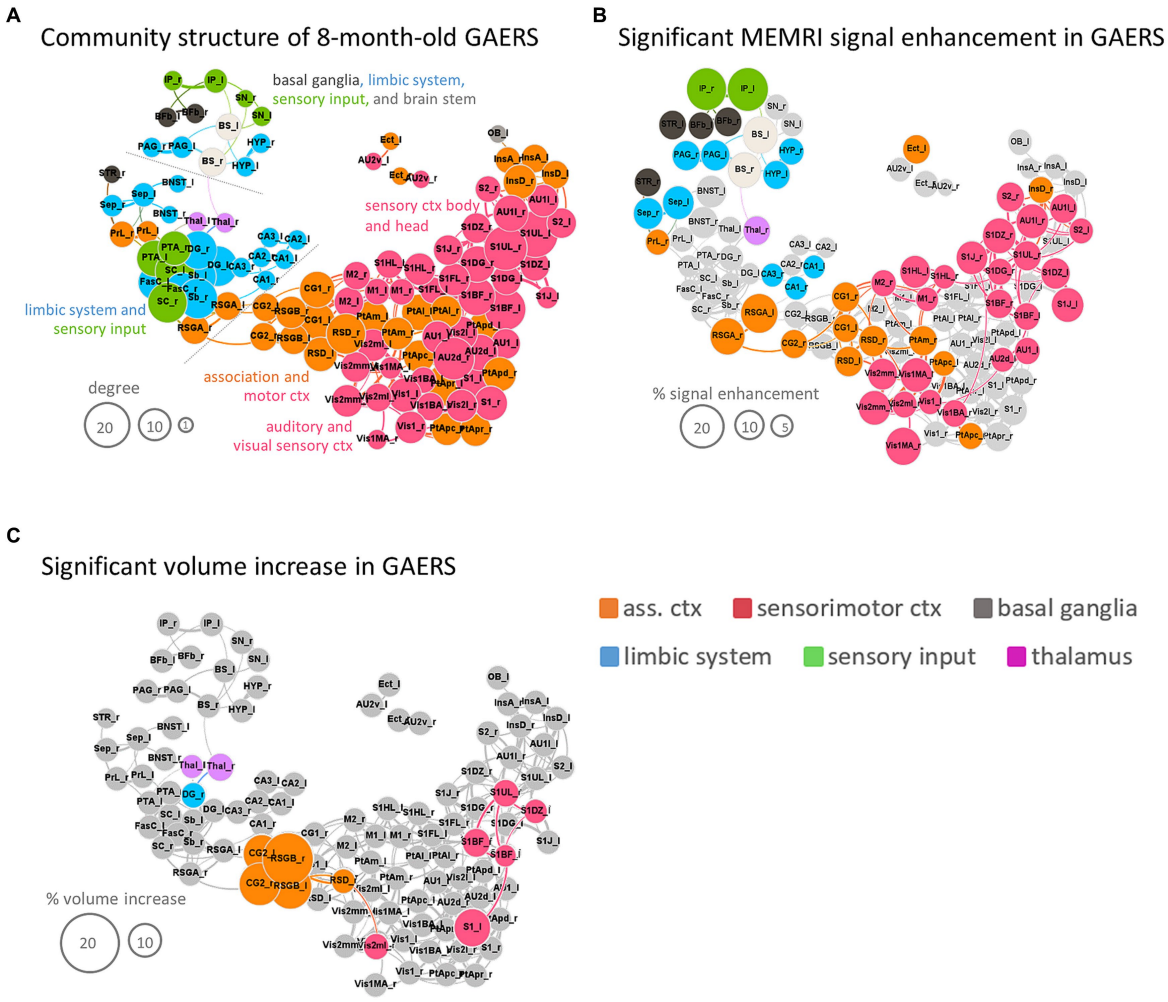
Synopsis. **(A)** Force-based network plot of community structure for GAERS based on analysis of 8 m rs-fMRI data with the RatSigma template at *k* = 6. **(B)** Same network plot as in **(A)**, but only highlighting regions with significant MEMRI hyperintensity. **(C)** Same network plot as in **(A)**, but only highlighting regions with significant volume increase. Node sizes scale with degree **(A)**, % signal enhancement **(B)** or % volume increase **(C)**, respectively. Colors indicate functional group affiliation (orange = association cortex, red = sensorimotor cortex, grey = basal ganglia, blue = limbic system, green = sensory input, pink = thalamus). Brain region abbreviations are listed in Supplementary Table S1.

To facilitate individual comparison of MEMRI with rs-fMRI data, the 8 m rs-fMRI data underwent a secondary graph analysis. A multiparametric analysis was conducted using the RatSigmaAtlas brain template, which consisted of 114 grey matter brain regions with fine-grained resolution of cortical and limbic regions and much coarser labelling of subcortical regions, in order to fully leverage the high resolution of MEMRI data. The resulting rs-network topology of GAERS at *k* = 6 (342 strongest connections) is displayed in Figure 7A. The network segregated again into 3 communities (indicated by dashed grey lines). One community was composed of sensorimotor (red) and association (orange) cortex regions, the second community united limbic regions (blue), sensory input regions (green) and thalamus (pink), and the third community included sensory input, limbic, basal ganglia (grey) regions and brain stem (white). Node size in Figure 7A scales with degree, highlighting the particularly strong FC within cortical brain regions.

Applying the RatSigmaAtlas brain template to MEMRI data revealed significantly higher manganese accumulation in approximately half of the analyzed brain regions in GAERS compared to NEC in line with TFCE maps (Figure 6) (i.e., 56 of 114 brain regions using the unpaired Student’s *t*-test, *p* < 0.01, FDR corrected). In particular, a significantly higher level of signal enhancement (highlighted in color in Figure 7B) was observed in the retrosplenial cortex (retrosplenial granular cortex part A, RSGA and retrosplenial dysgranular cortex, RSD), as well as in the insular (dysgranular insular cortex, InsD and cingulate cortex (primary, CG1, and secondary, CG2 cingulate cortex). Also the basal ganglia (striatum, STR, and basal forebrain, Bfb), the interpeduncular nucleus of sensory input region (IP), limbic regions such as the septum (Sep), hypothalamus (Hyp), periaqueductal gray (PAG) and subregions of the hippocampus (cornu ammonis, CA1 and CA3), and brain stem (BS) showed significantly higher manganese accumulation. The size of the nodes in Figure 7B is proportional to the percent difference in MEMRI signal between post-contrast GAERS and NEC.

#### Deformation-based volumetry

Total brain volumes were slightly greater in GAERS (WM 213 ± 4 mm^3^; GM 1586 ± 22 mm^3^; ventricle 32 ± 1 mm^3^) compared to NEC (WM 201 ± 5 mm^3^; GM 1537 ± 38 mm^3^; ventricle 32 ± 2 mm^3^) at 8 months of age (Student’s *t*-test, *p* < 0.001). Deformation-based analysis revealed significantly higher volumes (highlighted in color in Figure 7C) for secondary cingulate cortex and retrosplenial cortex (dysgranular cortex and granular cortex part B) in GAERS. Also, several regions of the sensory cortex (barrel field, dysgranular zone 0, trunk upper lip and primary and secondary visual cortex), as well as the limbic region dentate gyrus and the thalamus had higher volumes in GAERS.

#### Synopsis of 8 months data

Functional readouts and morphometry yielded consistent but partly complementary results. rs-fMRI identified more and stronger connections within the cortical community in GAERS, suggesting an important role of association and sensorimotor regions in the epileptic network. Notably, approximately half of the regions of the cortical community exhibited significant manganese accumulation. Specifically, secondary cingulate cortex and retrosplenial granular cortex part B also showed volume increase. Limbic regions exhibited strong rs-FC within the functional group and with association cortex and sensory input regions in GAERS. However, this high temporal synchronicity was associated with MEMRI hyperintensities only in PAG, Sep, hypothalamus (Hyp) and some hippocampal subregions (CA1 and CA3). The thalamus (right hemisphere) and the sensory input region interpeduncular nucleus (IP) and basal ganglia also exhibited significant MEMRI hyperintensity, but only the thalamus increased in volume. The basal ganglia, thalamus and IP showed relatively lower degree compared to cortical nodes.

## Discussion

### Major findings

Our longitudinal rs-fMRI data suggested that GAERS have a characteristic, disease-specific resting state-brain network, which appears to be fully developed in juvenile rats and differs from the network found in age-matched non-epileptic controls. MEMRI and volumetry data obtained from 8 months-old animals added further evidence for functional and morphological differences between GAERS and NEC.

### rs-fMRI

Graph theory analysis revealed that global network parameters were within the expected range and similar in GAERS and NEC, implying efficiently working brain networks in both strains. Across all timepoints, resting state-FC was consistently higher in GAERS. In line with our previous study (13), we found strong FC intra-cortically and between cortical, basal ganglia, and limbic regions in GAERS.

In contrast to the previous study, we now applied a different anesthesia protocol, using low-dose Isoflurane instead of Neurolept. By applying Isoflurane, GAERS were in a SWD-free state, under the same controlled anesthetic condition as NEC. We compared brain networks between GAERS and NEC, and expected the impact of low-dose Isoflurane to be similar and constant in both strains. Isoflurane-dose-dependent cortical segregation, and disruption of thalamo-cortical and subcortical FC have been reported (27, 36–38), and have been associated with loss of consciousness under anesthesia. We applied 1.1–1.2% Isoflurane and kept examination times short in order to preserve major brain networks (24, 39). The general network topology of GAERS and NEC in the present study was in agreement with rs-fMRI studies in naïve rodents, under Medetomidine (40, 41) and in the awake condition (42). In both strains, key nodes of the sensorimotor network, default mode network (DMN, Rs, and cg) and salience network (Ins and cg) formed a shared cortical community associated to varying extent with basal ganglia regions. Furthermore, a subcortical community consisting of limbic, sensory input, and thalamic regions was observed. In agreement with D’Souza (40), a small additional community with the sensory input region SC and with PAG was found.

We recognize that FC within the cortical community and with basal ganglia was rather high, whereas FC of thalamic nuclei within the brain network was less strongly represented. Despite the fact that these findings may indicate potential anesthesia effects, we detected significantly enhanced FC in GAERS compared to NEC in both age groups which seemed to be characteristic for the epileptic brain. We retrieved network components previously identified during absences in both animal models and human patients. The peri-oral region has long been shown to be the region of onset of absence seizures in WAG/Rij rats (20) and in GAERS (43). During ongoing seizures, SWD then occur generalized over the entire cortex. A high degree of cortico-cortical correlation has been reported in WAG/Rij rats under Neurolept sedation during epochs with and without SWDs, but not in non-epileptic control Wistar rats (14). Clemens et al. (44) interpreted the increased FC of the seizure onset area with the remaining cortical areas in humans as a state facilitating seizure propagation among the cortical network. The findings of Studer et al. indicate that in GAERS increased synaptic connectivity, especially through horizontal links from deep cortical neurons are critical in the neuronal synchronization in absence seizures (21). In absence epilepsy patients, involvement of the DMN and salience network (bilateral insular, anterior cingulate, and temporal–parietal cortices) has been recognized and has been associated primarily with cognitive dysfunction and disruption of attention and awareness during seizures (45–48). Other studies, using fMRI (49) or MEG (50, 51), additionally reported increased FC between sensorimotor and limbic regions and increased cortical FC between hemispheres (52). However, there are also some conflicting findings, such as reports of reduced interictal DMN (53) and attention network FC (45) in human patients compared to controls. Involvement of the basal ganglia in the remote control of absence seizures (54–56) has previously been suggested by pharmacological and electrophysiological studies in animal models of absence epilepsy (54–56). Consistently, a recent rs-fMRI study in CAE (57) found strong FC within the basal ganglia network and between basal ganglia nuclei and other brain regions.

Despite the occurrence of hundreds of absence seizures per day between 3 months and 8 months of age, we did not detect progressive FC changes in epileptic animals. Only in NEC we found age-related differences in FC. In adult NEC, NBS identified a component with statistically stronger intracortical connections, stronger FC between cortical regions and basal ganglia, and stronger FC between limbic and sensory input regions than in juvenile NEC. This aligns with findings in human studies (58, 59), which suggest an increase in the segregation of functional brain circuits with age. The shift is from stronger short-range connections in children, limited to primary sensory and motor brain regions, to stronger and more distinct patterns of long-range connections in adults, involving retrosplenial and insular cortex and subcortical regions. This shift has been linked to brain growth and expanding myelination, which enables faster information transmission (59). No such network differences were found between juvenile and adult GAERS. We speculate that the pathology in juvenile GAERS may have interfered with the normal shaping of circuitry during maturation. Aberrant functional connectivity has been implicated in neurodevelopmental disorders such as autism or attention deficit hyperactivity disorder (59).

When comparing between strains, NBS revealed a component with a higher number of significantly stronger connections in GAERS compared to NEC in both age groups, predominantly involving brain regions from association and sensorimotor cortex, from basal ganglia, and from limbic regions. Our NBS results support the notion that strong cortico-cortical FC reflect the strong synchronicity found during SWD activity. Basal ganglia regions, in particular the nucleus accumbens which showed high FC in our study are believed to play a central role in seizure control through their interplay with the striato-nigral pathway and the dopaminergic system (60). Enhanced expression of D3 dopamine receptor mRNA in the nucleus accumbens of adult GAERS in comparison to NEC has been observed (61). High FC within limbic regions and with cingulate cortex and insular cortex, also referred to as limbic cortex, on the other hand, may be related to social behavior deficits observed in GAERS (62). Sociability in rodents is thought to be mediated by neural circuits densely populated with T-type calcium channels and GAERS contain a missense mutation in the Cav3.2 T-type calcium channel gene. Henbid et al. observed reduced sociability in female GAERS (63).

Longitudinal neuroimaging data is more accessible from rat models of TLE. TLE models induce status epilepticus (SE) by focally lesioning the brain (e.g. with kainate injections). After an initial latency period, spontaneous recurrent convulsive seizures occur in the long-term (chronic phase). Histopathological examinations revealed massive local neuronal degradation and gliosis in this model. The severe pathology of TLE models compared to absence epilepsy models is associated with progressive brain network alterations. In a longitudinal rs-fMRI studies, analyzed through graph theory, Christiaen et al. (64) observed a decrease in global FC that was most significant during epileptogenesis, specifically occurring between 1 and 3 weeks post-SE. Afterwards, network connectivity remained unchanged. In addition to the finding that the maximum loss of hippocampal volume occurred 1 week post-SE, the authors concluded that alterations in network topology resulted from neuronal tissue loss. One intriguing finding from that study was a negative correlation between early shifts in network topology and later seizure frequency, suggesting that a minimal level of organization is necessary for seizures emergence. In agreement, Bertoglio et al. (65) reported widespread hyposynchrony at 2 weeks post-SE in the same model. However, a significant increase in network synchronicity occurred between 2 to 4 weeks post-SE, suggesting potential network reinforcement during seizure recurrence. Gill et al. (66) also observed elevated intra- and interhemispheric connectivity, particularly within the DMN and between limbic and DMN structures, 5 weeks after SE in animals, with postmortem histologically confirmed neuronal damage. Li et al. (67) detected increased FC in the sensorimotor cortex and limbic regions at 10 days post-SE, which was found to be a predictive marker for epileptogenesis. This was confirmed through longitudinal EEG recordings between the ages of 2 and 4 months. In these rodent models, progressive brain network alterations towards lower FC appeared to occur in temporal sequence with TLE-related macroscopic brain alterations. Once the epilepsy phenotype is established, network connectivity that is either stable or increased is observed. The brain regions involved in this network include cortical, basal ganglia, and limbic regions, which are of importance also in our current study of absence epilepsy.

### MEMRI

We utilized MEMRI as an independent readout of FC for 8 months-old GAERS and NEC. Our findings demonstrated that GAERS exhibited higher overall brain signal enhancement, which indicated higher integrated manganese accumulation in epileptic brains and is consistent with enhanced overall rs-FC. TFCE-maps revealed the highest significant bilateral differences in the retrosplenial cortex, medial entorhinal cortex, brain stem and interpeduncular nucleus, as well as in the right primary visual cortex and somatosensory cortex. Region-based analysis revealed significantly higher signal intensities in approximately half of the analyzed brain regions in GAERS compared to NEC. Both MEMRI and rs-fMRI consistently showed higher brain activity in GAERS compared to NEC in cortical key components of sensorimotor network, DMN (retrosplenial and cingulate cortex) and salience network (insular cortex). In subcortical regions, functional readouts showed less distinct resemblance. MEMRI hyperintensities were prominent in interpeduncular nucleus, limbic regions septum, hypothalamus and PAG, while rs-fMRI displayed relatively higher connectedness in sensory input regions and various hippocampal subregions (dentate gyrus and fasciola cinereum). Both rs-fMRI and MEMRI provided consistent but partly complementary information. rs-fMRI took a “snapshot” of synchronous, spontaneous, neuronal activity in the anesthetized animal. However, fMRI only provides an indirect readout of neuronal activity. The temporal representation of brain activity by fMRI is inherently blurry, because the hemodynamic response is slow (range of seconds) compared to neuronal activity (tens of millisecond range) and the time resolution of the rs-fMRI sequences is in the range of seconds. MEMRI hyperintensity reflected the integrated neuronal activity (with and without seizures) over a week in behaving 8 months-old animals. Recently, the role of astrocytes as a sink for manganese has been discussed (68), but it should be noted that MRI is unable to distinguish astrocytes from other cells. Enhanced manganese accumulation was observed during post-stroke inflammatory processes and was associated with activated microglia and reactive astrocytes (69). Neuronal loss as a prerequisite of local brain inflammation was not found in GAERS (70), but reactive astrogliosis coupled with altered cytokine expression has been observed in GAERS and was linked to epileptogenesis (71).

Several previous publications have demonstrated the high sensitivity of MEMRI to map neuronal activity. In naïve animals, both natural (72) and artificial stimuli (73) elicited local hyperintensities following the systemic administration of MnCl_2_. Eschenko et al. (74) reported patterns of running-induced T1-weighted MRI signal enhancement resulting from increased Mn^2+^ accumulation in activated brain regions in voluntarily running rats, compared to the sedentary controls. Another study found that MEMRI can identify brain regions linked to food intake, reward/addiction and locomotor activity in a rat model of hyperphagia (75). However, in other studies examining TLE, MEMRI contrast was not sensitive to prolonged seizure activity during the chronic phase (76, 77). Dedeurwaerdere et al. (78) even reported an inverse correlation between seizures and MEMRI signal after intraventricular MnCl_2_ injection in the chronic epileptic phase. Only Alvestad et al. (79) identified MEMRI hyperintensities in the entorhinal cortex and amygdala after subcutaneous injection of manganese in a TLE study during the chronic phase. In our study, we observed overall MEMRI hyperintensities at maximum moderate levels. Absence seizures are characterized by highly synchronized neuronal activity generalized across the cortex, but the degree of activity may be lower than anticipated, especially when considering the ratio of total seizure time (ictal) to interictal time. Furthermore, a recent EEG-fMRI study in GAERS (80) revealed long-lasting decreased seizure-associated activity in most cortical and thalamic neurons. The authors linked their findings to the loss of consciousness during seizures.

### Volumetry

At 8 months of age, we observed higher brain volumes in GAERS as compared to age-matched NEC. Boullieret et al. (81) also found increased volumes in GAERS compared to NEC in amygdala and cortex already at 3 months of age. But, Kirazli et al. (82) reported reduced thickness in auditory and visual cortices, and striatum, and overall brain size in GAERS at 1 and 2 months of age based on a histological study. Notably, in that study, normal Wistar and GAERS did not show significant volumetric differences at P10, and the authors speculated that GAERS are ordinary at that early age. The clinical findings remain conflicting and differ by region. In agreement with the current study, a voxel-based morphometry meta-analysis (83) confirmed augmented gray matter volumes in the bilateral medial frontal gyrus and anterior cingulate. Additionally, an increased thickness of the posterior medial cortex, a region linked to the DMN, was reported in patients with newly onset CAE (84). Some researchers have observed widespread cortical thinning in patients with absence seizures (85), while others have found no cortical thinning with age (86). Thalamic volumes have been found to be increased (87), but several CAE studies have reported thalamic and caudate atrophy (88–91).

### General conclusion

rs-fMRI, MEMRI and volumetric data collectively suggest the importance of cortical networks in GAERS, which correlates with an increased fronto-central connectivity in human absence epilepsy patients. Our findings also verify involvement of basal ganglia and limbic regions. The network differences between GAERS and NEC are observed already in juvenile animals. The findings by Jarre et al. (92) who identified initial oscillatory discharges in the primary somatosensory cortex as early as in the third week of life in GAERS by cortical LFP measurements indicate that the formation of the epileptic network occurs before 3 months of age. In clinical studies, Davis et al. (93) found increased cortical EEG-connectivity during sleep already in infants with disrupted early brain development at the cellular level in tuberous sclerosis complex who later developed epilepsy. These authors speculated that neurological diseases occurring during critical stages of brain maturation may affect normal neurodevelopment. Our preclinical study indicates that an epilepsy-related network is already present very early in neurodevelopment and that this early condition shapes dynamic brain function more than chronic absence seizures. Clinical studies to further test this hypothesis are recommended.

## Data availability statement

The raw data supporting the conclusions of this article will be made available by the authors, without undue reservation.

## Ethics statement

The animal study was approved by Landesamt für Natur, Umwelt und Verbraucherschutz, North Rhine Westphalia, Germany. The study was conducted in accordance with the local legislation and institutional requirements.

## Author contributions

LW: Conceptualization, Data curation, Formal analysis, Investigation, Methodology, Supervision, Validation, Visualization, Writing – original draft, Writing – review & editing. LH: Formal analysis, Investigation, Validation, Visualization, Writing – original draft, Writing – review & editing. JP: Formal analysis, Writing – review & editing. LK: Formal analysis, Writing – review & editing. HL: Formal analysis, Writing – review & editing, Investigation. AL: Resources, Writing – review & editing, Funding acquisition. TB: Resources, Writing – review & editing. AH: Formal analysis, Methodology, Software, Visualization, Writing – review & editing. CF: Funding acquisition, Project administration, Resources, Supervision, Writing – review & editing.
