# Supplementary material for: Epilepsy-related functional brain network alterations are already present at an early age in the GAERS rat model of genetic absence epilepsy
Source: Front Neurol. 2024 Mar 11;15:1355862. doi: 10.3389/fneur.2024.1355862 (PMC10961455; doi:10.3389/fneur.2024.1355862)

Epilepsy-related functional brain network alterations are already present at an early age in the GAERS rat model of genetic absence epilepsy

Lydia Wachsmuth[1†] , Leo Hebbelmann[1†] , Jutta Prade[3], Laura C. Kohnert3, Henriette Lambers[1], Annika Lüttjohann[2], Thomas Budde[2], Andreas Hess[3,4,5], Cornelius Faber[1*]

Supplemental Material

Suppl. table 1: List of brain regions. (**A**) The MagnAn atlas, applied for longitudinal rs-fMRI data analysis, contained 38 bihemispheric and 4 central grey matter brain regions (highlighted in bold). (**B**) The Rat Sigma atlas, applied for analysis of 8-month-rs-fMRI data, high-resolution MEMRI data and for volumetry, contained 57 bihemispheric grey matter regions. The latter subdivided cortex and hippocampus into many subregions, but provided only a limited number of subcategories for the subcortical brain. Colors indicate functional group affiliation. The hemisphere is indicated by the appendix _l or _r in the manuscript.

table on next page

A MagnAn atlas

association cortex	Cg	Cingulate Cortex
	Ins	Insular Cortex
	PtA	Parietal Association Cortex
	RS	Retrosplenial Cortex
	TeA	Temporal Association Cortex
sensorimotor cortex	S1HL	Primary Somatosensory Cortex Hind Limb
	S1r	Primary Somatosensory Cortex rest
	S2	Secondary Somatosensory Cortex
	M1	Primary Motor Cortex
	M2	Secondary Motor Cortex
basal ganglia	Acb	Nucleus Accumbens
	Cl	Claustrum
	CPu	Striatum
	GP	Globus Pallidus
	VP	Ventral Pallidum
limbic system	BST	Bed Nucleus of Stria Terminalis
	**CoM**	**Corpora Mammillaria**
	DB	Nuclei of Diagonal Band
	Hb	Habenuli
	Hip	Hippocampus
	HT	Hypothalamus
	Sep	Septum
	SLEA	Sublenticular Extended Amygdala
	ZI	Zona Incerta
	**PAG**	**Periaqueductal gray**
sensory input	PTA	Pretectal Area
	Re	Red Nucleus
	SC	Superior Colliculus
	Teg	Tegmental Nuclei
	Pn	Pontine nucleus
	**R**	**Raphe Nucleus**
	VTA	Ventral Tegmental Area
thalamus	Ant	Anterior Thalamic Group
	LG	Lateral Geniculate Nucleus
	LP	Lateral Posterior Thalamic Nucleus
	MG	Medial Geniculate Nucleus
	MT	Medial Thalamus
	Po	Posterior Thalamic Nuclear Group
	Rt	Reticular Thalamic Nucleus
	VM	Ventromedial Thalamic Nucleus
	VPL_VPM	Ventral Postolateral/Posteromedial Thalamic Nucleus
	**PV**	**Paraventricular Thalamic Nucleus**

B RatSigma atlas

association cortex	Lateral Parietal Associative Cortex	PtAl
	Medial Parietal Associative Cortex	PtAm
	Parietal Cortex Postero Caudal Part	PtApc
	Parietal Cortex Postero Dorsal Part	PtApd
	Parietal Cortex Postero Rostral	PtApr
	Ectorhinal Cortex	Ect
	Agranular Insular Cortex	InsA
	Dysgranular Insular Cortex	InsD
	Posterior Agralunar Insular Cortex	InsAp
	Primary Cingular Cortex	CG1
	Secondary Cingular Cortex	CG2
	Retrosplenial Dysgranular Cortex	RSD
	Retrosplenial Granular Cortex Part A	RSGA
	Retrosplenial Granular Cortex Part B	RSGB
	PreLimbic System	PrL
sensorimotor cortex	Primary Somatosensory Cortex Barrel field	S1BF
	Primary Somatosensory Cortex Dysgranular	S1DG
	Primary Somatosensory Cortex Dysgranular Zone 0	S1DZ
	Primary Somatosensory Cortex Forelimb	S1FL
	Primary Somatosensory Cortex Hindlimb	S1HL
	Primary Somatosensory Cortex Jaw	S1J
	Primary Somatosensory Cortex	S1
	Primary Somatosensory Cortex Upperlips	S1UL
	Secondary Somatosensory Cortex	S2
	Lateral Primary Auditory Cortex	AU1l
	Primary Auditory Cortex	AU1
	Secondary Auditory Cortex Dorsal Part	AU2d
	Secondary Auditory Cortex Ventral Part	AU2v
	Lateral Secondary Visual Cortex	Vis2l
	Medio Lateral Secondary Visual Cortex	Vis2ml
	Medio Medial Secondary Visual Cortex	Vis2mm
	Primary Visual Cortex Binocular Area	Vis1BA
	Primary Visual Cortex	Vis1
	Primary Visual Cortex Monocular Area	Vis1MA
	Primary Motor Cortex	M1
	Secondary Motor Cortex	M2
basal ganglia	Striatum	STR
	Basal Forebrain Region	BFb
limbic system	Septal Region	Sep
	Cornu Ammonis 1	CA1
	Cornu Ammonis 2	CA2
	Cornu Ammonis 3	CA3
	Dentate Gyrus	DG
	Fasciola Cinereum	FasC
	Subiculum	Sb
	Amygdalopiriform Cortex	AMpir
	Bed Nucleus of the Stria Terminalis	BNST
	Hypothalamic Region	HYP
	Medial Entorhinal Cortex	Entm
	Periaqueductal Gray	PAG
sensory input	Interpeduncular Nucleus	IP
	Pretectal Region	PTA
	Superficial Gray Layer of the Superior Colliculus	SC
	Substantia Nigra	SN
thalamus	Thalamus	Thal
	Olfactory Bulb	OB
	Brainstem	BS

- association cortex
- sensorimotor cortex
- basal ganglia
- limbic system
- sensory input
- thalamus

Suppl. figure 1: Mean correlation matrices of juvenile and adult NEC (**A, B**) and GAERS (**C, D**), respectively. Brain regions are arranged according to affiliation with functional group and hemisphere, as indicated in the top row. Upper triangles of adjacency matrices display FDR-corrected Pearson r-values between brain regions. Lower triangles display 240 strongest Pearson r-values at a network density k = 6. Diagonals represent the interhemispheric connections between regions.

figure on next page

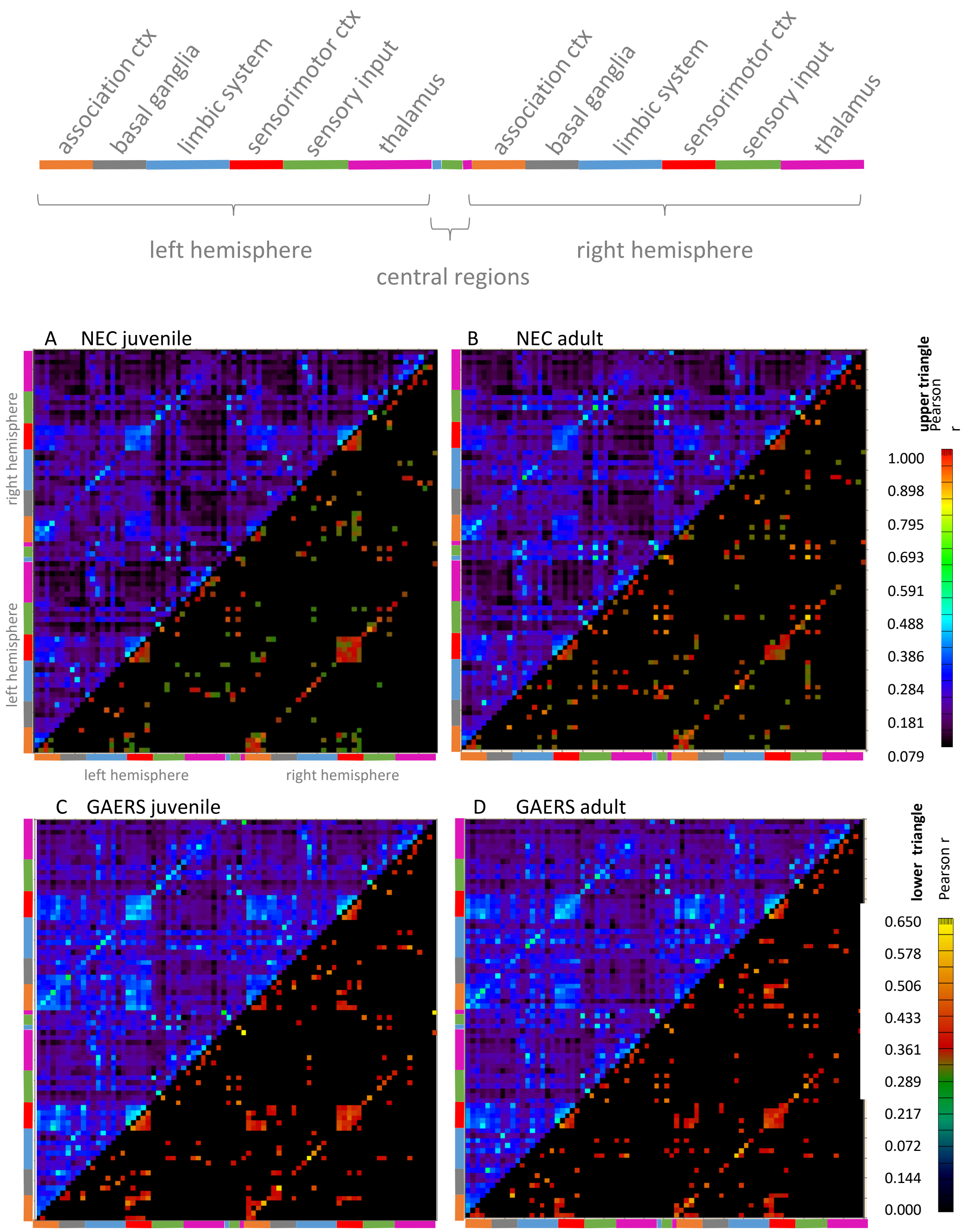
association ctx
basal ganglia
limbic system
sensorimotor ctx
sensory input
thalamus
left hemisphere
central regions
right hemisphere
A NEC juvenile
B NEC adult
C GAERS juvenile
D GAERS adult
upper triangle
Pearson r
1.000
0.898
0.795
0.693
0.591
0.488
0.386
0.284
0.181
0.079
lower triangle
Pearson r
0.650
0.578
0.506
0.433
0.361
0.289
0.217
0.072
0.144
0.000

Suppl. figure 2: Local node parameters. Group-averaged degree for both juvenile and adult GAERS (**A**) and NEC (**B**) sorted as per functional group (color) and hemisphere (left-right). The brain regions within the top 20 degree ranks are highlighted in bold colors (orange = association cortex, red = sensorimotor cortex, grey = basal ganglia, blue = limbic system, green = sensory input, pink = thalamus).

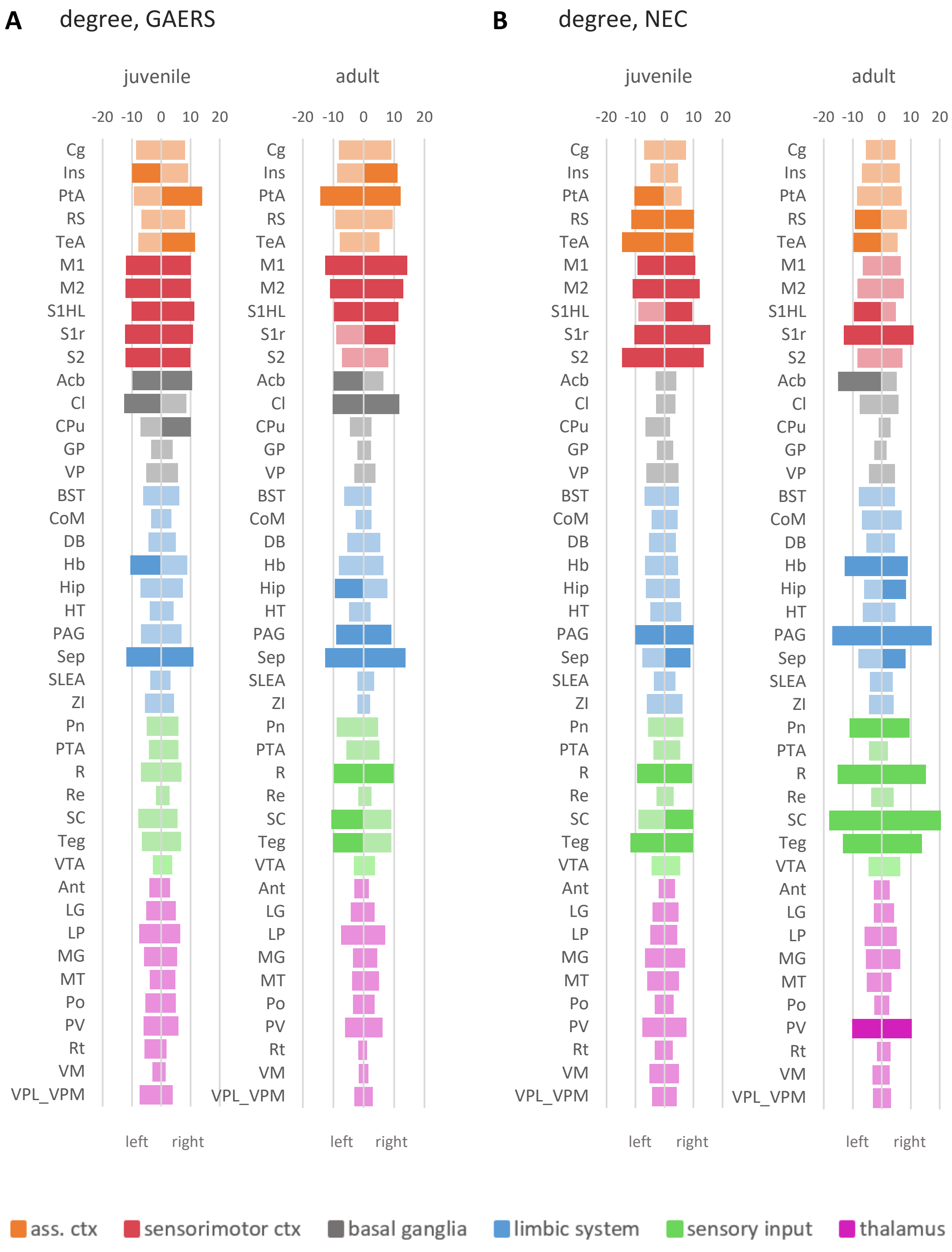

Suppl. figure 3: Local node parameters. Radar plots display strength (**A**), degree (**B**), clustering coefficient (**C**), path length (**D**), betweeness (**E**), and hubscore (**F**) averaged by hemisphere and age group. Light blue lines show values for brain regions in NEC, light orange lines show values for brain regions in GAERS. Asterisks indicate significant differences between strains (Posthoc Mann Whitney U * = $p < 0.05$, ** = $p < 0.01$). Brain region abbreviations are listed in suppl. table 1. A summary of the results of variance analyses and posthoc tests are reported in suppl. table 3.

figure on next page

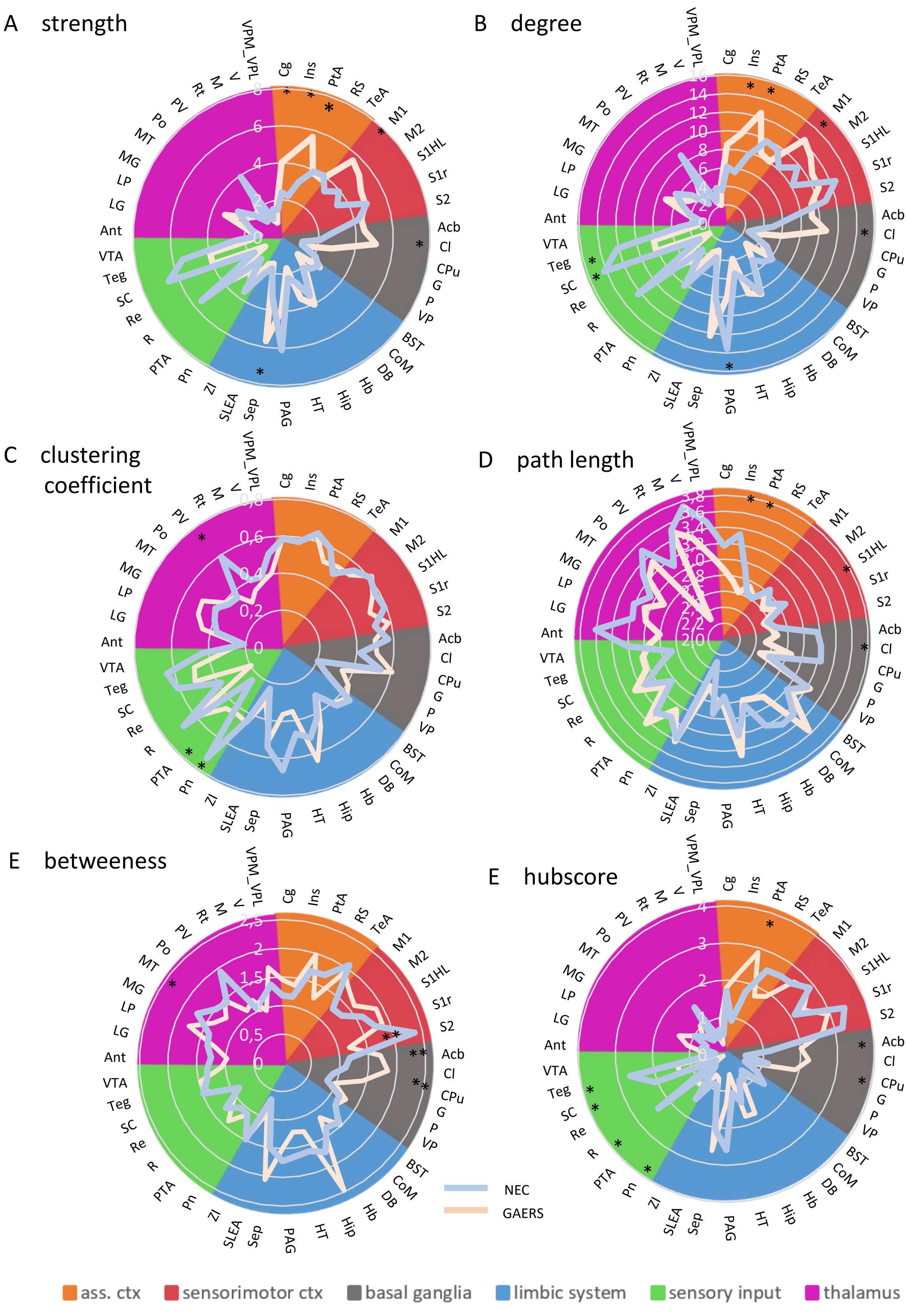

A strength
B degree
C clustering coefficient
D path length
E betweeness
E hubscore
VPM_VPL
Cg
Ins
PtA
RS
TeA
M1
M2
S1HL
S1r
S2
Acb
Cl
CPu
G
P
VP
BST
CoM
DB
Hb
Hip
HT
PAG
Sep
SLEA
ZI
Pn
PTA
R
Re
SC
Teg
VTA
Ant
LG
LP
MG
MT
Po
PV
Rt
M
V
NEC
GAERS
ass. ctx
sensorimotor ctx
basal ganglia
limbic system
sensory input
thalamus

Suppl. Figure 4: Group-averaged MEMRI data. Mean signal intensity maps were acquired after 7 days of manganese accumulation in (**A)** NEC and (**B)** GAERS. Selected axial slices from mean 3D T1w data sets are shown. Warm colors indicate higher signal intensity in arbitrary units. Note that maximum signal intensity is higher in GAERS (**B**).

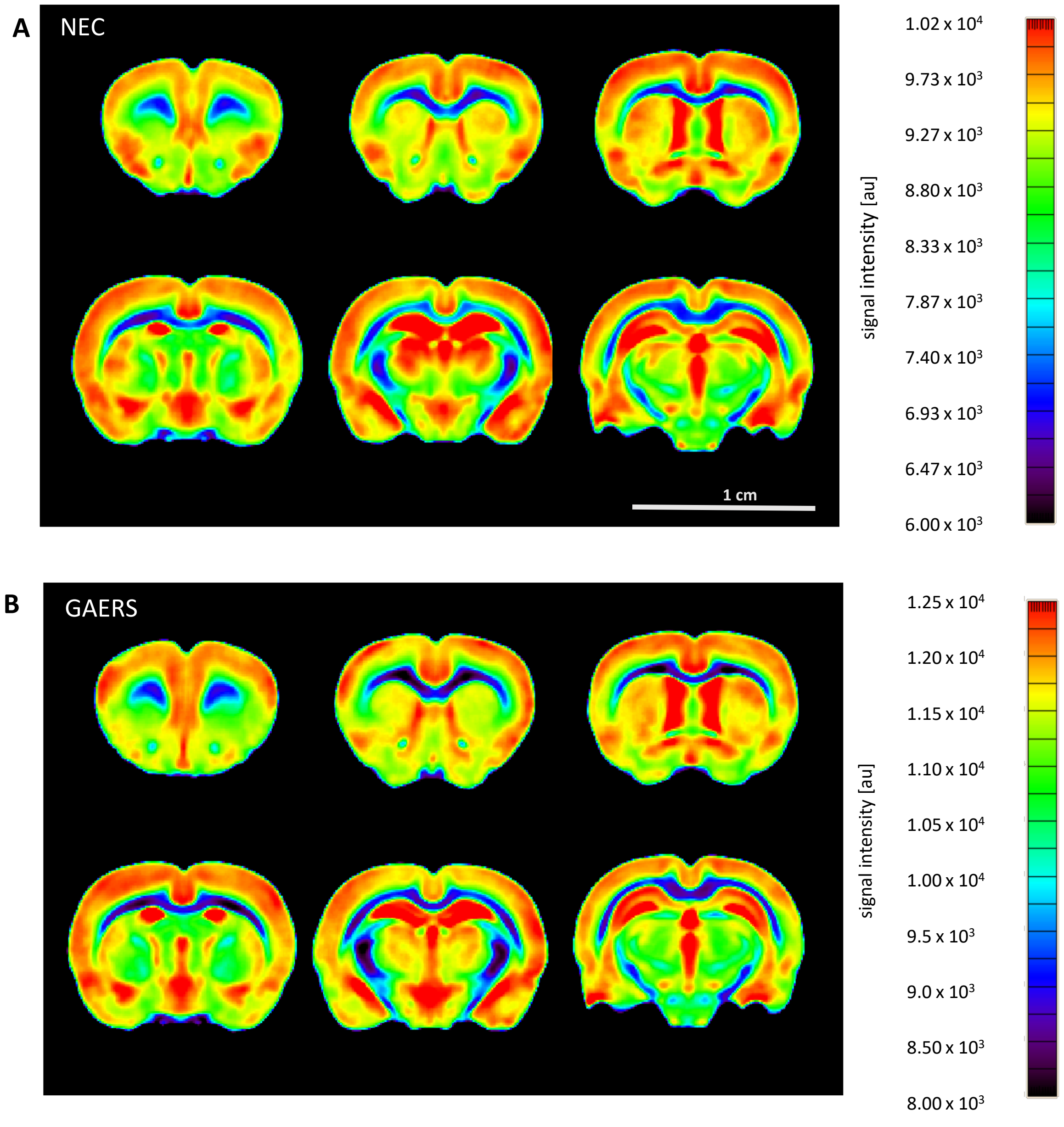

Supplement: Supplementary file 1 [file Data_Sheet_1.pdf]
